# Umbilical cord serum concentrations of perfluorooctane sulfonate, perfluorooctanoic acid, and the body mass index changes from birth to 5 1/2 years of age

**DOI:** 10.1038/s41598-021-99174-3

**Published:** 2021-10-05

**Authors:** Takanobu Horikoshi, Tomoko Nishimura, Yoko Nomura, Toshiki Iwabuchi, Hiroaki Itoh, Takumi Takizawa, Kenji J. Tsuchiya

**Affiliations:** 1 Department of Pediatrics, Gunma University Graduate School of Medicine, Maebashi, Japan; 2 Research Center for Child Mental Development, Hamamatsu University School of Medicine, Hamamatsu, Japan; 3 United Graduate School of Child Development, Hamamatsu University School of Medicine, Hamamatsu, Japan; 4 The City University of New York, New York, NY USA; 5 The Icahn School of Medicine at Mount Sinai, New York, NY USA; 6 Department of Obstetrics and Gynaecology, Hamamatsu University School of Medicine, Hamamatsu, Japan

**Keywords:** Environmental chemistry, Intrauterine growth, Obesity, Paediatric research

## Abstract

Prenatal exposure to perfluoroalkyl substances (PFAS) has been reported to affect body weight from birth to childhood, but the results remain inconclusive. We investigated whether umbilical cord blood concentrations of perfluorooctane sulfonate (PFOS) and perfluorooctanoic acid (PFOA) are associated with children’s risk trajectory for obesity. 600 children were randomly selected from the Hamamatsu Birth Cohort for Mothers and Children (HBC study) and their umbilical cord serum PFAS concentrations were quantified. Participants underwent BMI measurements at ages 1, 4, 10, 18, 24, 32, 40, 50, and 66 months. Growth curve modeling with random intercept was performed with standardized BMI as outcome variable. PFOS was negatively associated with standardized BMI (β =  − 0.34; *p* = 0.01), with a marginally significant interaction with the child’s age (β = 0.0038; *p* = 0.08). PFOA was negatively associated with standardized BMI (β =  − 0.26, 95% CI − 0.51, 0; *p* = 0.05), with a significant interaction with the child’s age (β = 0.005; *p* = 0.01). Stratified analysis by sex revealed that these effects were significant only among girls. Prenatal exposure to PFAS initially was associated with lower standardized BMI during infancy, but this effect dissipated over time and reversed in direction during later childhood. The effects of prenatal PFAS on higher standardized BMI is stronger in girls.

## Introduction

Perfluoroalkyl substances (PFAS), including perfluorooctane sulfonate (PFOS) and perfluorooctanic acid (PFOA), are a group of synthetic chemicals which have a totally fluorinated carbon chain and a terminal acid^1^. PFAS have some unique properties, including water and oil repellency and extremely high thermal and chemical stability^2^. Owing to these properties, PFAS have been used for a variety of products such as carpets, textiles, clothes, cookware, food packaging, and fire-fighting foam since the 1950s^3,4^. In the 1990s, PFOS and PFOA were detected in the blood of the general human population^5^. In 2000, the 3 M company, a principal manufacturer of PFAS, announced it would discontinue producing PFOS and related compounds^6^. In 2009, PFOS was included in Annex B of the Stockholm Convention on Persistent Organic Pollutants, indicating the initial recognition of PFOS as toxic, and it was subsequently banned. Despite the ban, PFOS remained in human serum due to its high persistency in the environment and long serum elimination half-lives (5.4 years for PFOS and 3.8 years for PFOA)^7^. PFAS accumulate in the blood primarily by binding to albumin^8^, and can cross the placental barrier^9^. They act as endocrine disrupters, affecting estrogen, thyroid hormone, and glucocorticoids^10–13^, and may influence the peroxisome proliferator-activated receptor (PPAR) α that mediates lipid metabolism in adipocytes^14^. Studies in rodents have shown that prenatal exposure to PFOA causes neonatal death, developmental delay, and postnatal growth deficits^15,16^.

Thus far, reported associations between PFAS and body weight from birth to childhood are still elusive. Many epidemiologic studies on human subjects have demonstrated an association between prenatal PFAS exposure and either a null or small reduction in birthweight^17–20^. During childhood, studies have shown that increased prenatal exposure to PFAS was associated with a greater risk of obesity or weight gain^21–24^, while others have shown no notable associations^25,26^. A recent meta-analysis found that exposure to PFOA in early life is associated with an increased risk for childhood adiposity^27^. Chen et al. showed that higher umbilical cord plasma PFOS concentration was associated with lower birthweight and height, but these effects diminished as children aged^28^. They further showed the effects were more obvious among girls. However, as the authors acknowledged, considerable attritions during the follow-up period and their analytical plan which lacks adjusting for gestational age or parity prevented them from drawing a clear conclusion. To clarify and validate this line of investigation, capitalizing on the available longitudinal and frequent assessments and very low attrition rate in the Hamamatsu Birth Cohort for Mothers and Children (HBC Study)^29^, we set out to evaluate the association between prenatal exposure to two most widely studied PFAS, PFOS and PFOA, and the trajectory of BMI from birth to early childhood (i.e., 5 1/2 years) with adjustment of a priori determined important potential confounders.

## Methods

### Study population

The study population is derived from a birth cohort study in Hamamatsu, Japan, the HBC Study, with data from mothers (n = 1138) and their children (n = 1258) born between December 2007 and March 2012. A majority (98.8%) of enrolled mothers were Japanese. All mothers were enrolled at either one of the two research sites (Hamamatsu University Hospital and Kato Maternity Clinic) between November 2007 and March 2011 during the first or second trimester of pregnancy and gave birth at Hamamatsu University Hospital. As previously reported^30^, enrolled mothers and children were representative of the general Japanese population with regard to age and socioeconomic status of the families, and the child’s birthweight and gestational age at birth, compared to Japanese government’s national statistics^29,31^. The details of the parent study have been described elsewhere^29^.

**Figure 1:**
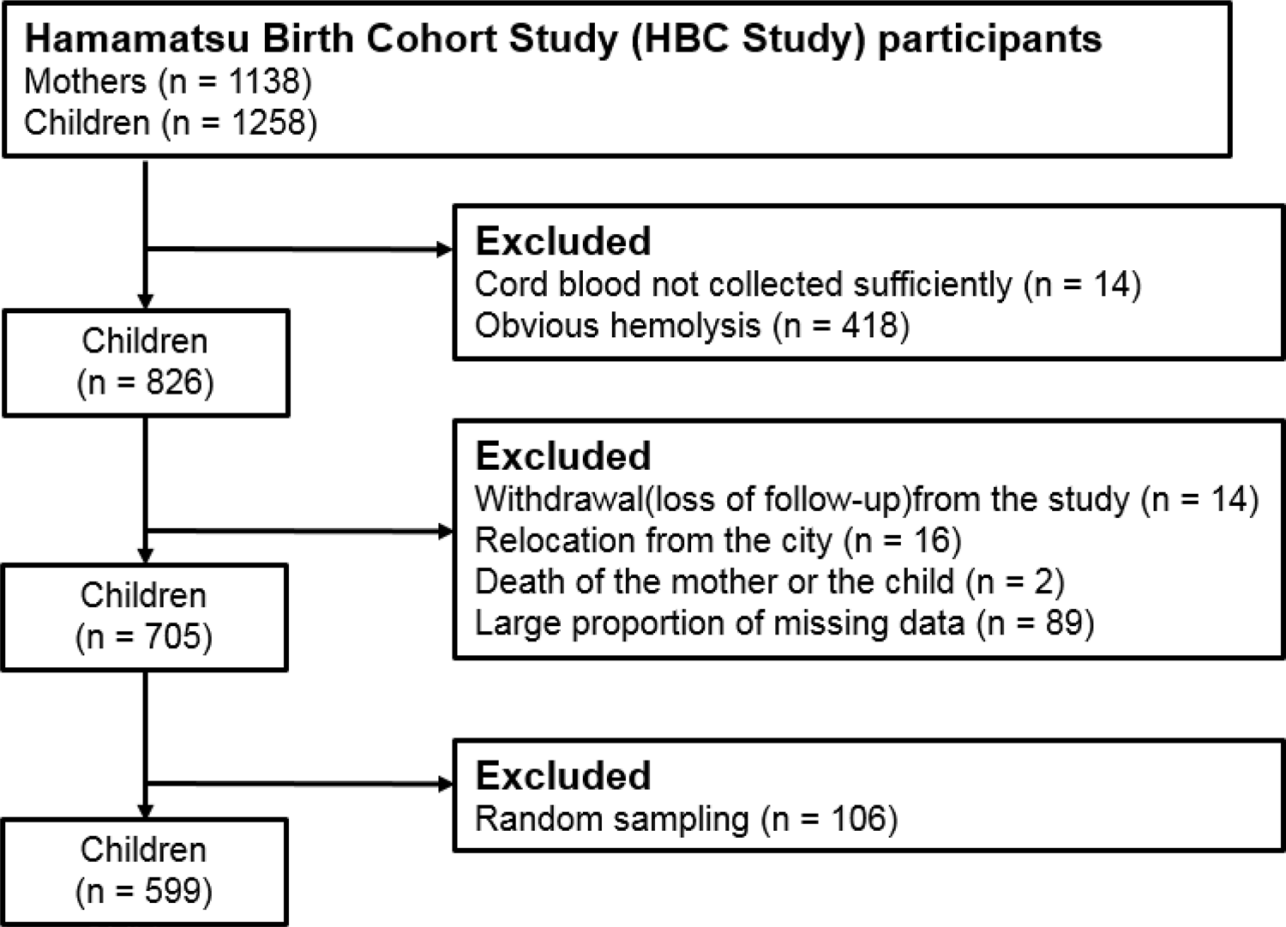
Flow chart of the participant selection.

Of the 1258 live births, umbilical cord venous blood was collected from 1244. Among the 1244 available samples, constrained by available funds, 600 were randomly selected for assays. Of those 600, 1 case was excluded due to the child having developed serious developmental delays (Fig. 1). Two more were excluded as the assay values were beyond the limit of detection (LOD) of the assay used to measure PFOS and PFOA concentrations (see below), leaving 597 samples.

### Exposure

We used umbilical cord serum PFOS and PFOA concentrations as the measurement of prenatal exposure. Umbilical cord blood (UCB) was collected immediately after birth and placed at room temperature for 30 min. It was then centrifuged, and the serum layer was transferred to Eppendorf tubes and stored at − 80 °C freezer until analysis. PFOS and PFOA concentrations were analyzed at Shimadzu Techno-Research, Inc. (Kyoto, Japan) using a high-performance liquid chromatography (HPLC) (Prominence; Shimadzu Corporation) coupled with a tandem mass spectrometer (MS/MS) (API 4000; AB Sciex) followed by solid phase extraction. Detailed methods are described in supplemental document.

### Outcomes

We used BMI to evaluate children’s physical status. Between January 2008 and September 2017, the participating children were invited for assessments at Hamamatsu University Hospital at age 1, 4, 10, 18, 24, 32, 40, 50, and 66 months. At each assessment, the children’s weight was measured to the nearest 0.1 kg and height to the nearest 0.1 cm. BMI was transformed into standard deviation scores (SDS) against the data from the Japanese Society for Pediatric Endocrinology^32^. This standardized BMI (BMI SDS) was used as our outcome variable.

### Statistical analysis

Statistical analyses were performed using STATA version 14.2 (StataCorp LP, Texas, USA) and Mplus 7.4 (www.statmodel.com). PFOS and PFOA concentrations were log10 transformed to reduce the influence of outliers and right-skewed distribution. Growth curve modeling with random intercept was used to analyze association of cord serum PFAS levels with a series of BMI SDS records over time as a best-fit line for each participant, considering individual variation of BMI SDS at birth (i.e. at baseline). Similarly, interaction between PFOS or PFOA concentration and months of age was calculated and entered into the models to estimate the slope of the best-fit line of BMI SDS over time. Thus, estimated intercept parameters represent BMI SDS at birth and estimated slope parameters indicate change in PFOS or PFOA concentration per month throughout the observation period. PFOS and PFOA concentrations were tested both dimensionally and categorically (i.e., tertiles) to assess potential non-linear association between PFAS concentration and child body weight^21^. Based on our prior research findings^21,23,25^, variables that were associated with both PFAS concentrations and child body weight were included as covariates in the adjusted model, including maternal age at delivery, maternal BMI before pregnancy, maternal education, household income, maternal smoking during pregnancy, parity, gestational age, and duration of breast feeding. Since a recent study suggests that the magnitude of the association between PFAS and BMI differed between males and females^28^, we conducted an additional analysis by sex to see whether the associations under investigation can be observed for both sexes.

Of the 597 infants included, 30 (5.0%) infants had 5 or more missing data on BMI, and 18.8% had at least one missing BMI data across 9 observations between 1 and 66 months. We leveraged the central methodological strength of growth curve modeling and generated estimates for the missing data using maximum likelihood (ML) method. ML involves the computation of a case-wise likelihood function using all observed variables for a particular case, while including partially complete cases to estimate parameters for the missing data. Monte Carlo studies have shown that ML involves less restrictive assumptions about patterns of missing information and yields unbiased parameter estimates, increases the efficiency of parameter estimates, and eliminates bias in estimation arising from listwise or pairwise deletion and mean substitution of cases^33–35^.

### Ethical issues

This study was approved by the Hamamatsu University School of Medicine and University Hospital Ethics Committee. Written informed consent was obtained from all mothers for her own and her child’s participation. All research was performed in accordance with the relevant guidelines and regulations.

## Results

534 mothers and 597 children participated in this study (Table 1). There were no significant demographic differences between those included and those excluded except for in birth weight (*p* = 0.05) and parity (*p* = 0.009) (see Supplementary Table 2 and Supplementary Table 3, Supplementary Information).

**Table 1:** Characteristics of the mothers (n = 534) and children (n = 597) participating in the study.

Characteristics (n = 597)	Median (IQR)	N (%)
Maternal age at birth (years)	31.8 (28.1, 35.3)	
Maternal pre-pregnancy BMI (kg/m^2^)	20.3 (18.8, 22.5)	
Household income (10,000 yen/year)	550 (420, 700)	
Maternal education (years)	14 (12, 16)	
Gestational age (weeks)	39.14 (38.14, 40.0)	
Birthweight (g)	2982 (2699, 3246)	
Duration of breastfeeding (months)	10 ( 4, 14 )	
**Sex**		
Boys		310 (52)
Girls		287 (48)
**Parity**		
Primiparous		274 (46)
Multiparous		323 (54)
**Prenatal exposure to smoking**		
Yes		41 (7)
No		556 (93)

Mean cord serum concentrations (ng/mL) of PFOS and PFOA were 1.38 and 1.39, respectively (Table 2). In the analysis using a dimensional measure of log10-transformed concentrations of PFOS and PFOA, there was a significant effect of PFOS concentration on the intercept of BMI SDS in both unadjusted and adjusted analyses, indicating that a higher PFOS concentration was associated with a lower BMI SDS at birth (Table 3). Meanwhile, the interaction between PFOS concentration and child’s age, representing the slope of the individual best-fit line, was only marginally significant. Associations were clearer with PFOA. Specifically, higher PFOA concentration was associated with lower BMI SDS at birth in the adjusted model. Furthermore, there was a significant positive interaction between PFOA concentration and child’s age on BMI SDS, representing the positive slope (increase in the BMI SDS as they grow), in both unadjusted and adjusted model. In the analysis by sex, only among girls higher PFOS and PFOA concentrations were associated with lower BMI SDS at birth and had significant positive interactions with child's age on BMI SDS (positive slope) indicating that greater levels of PFOS and PFOA are associated with greater increased in the BMI SDS as the child grows.

**Table 2:** Measured concentrations of cord serum PFOS and PFOA (ng/mL).

	Mean	Standard deviation	Range	Low tertile	Middle tertile	High tertile
PFOS	1.38	0.21	0.21, 7.10	0.21, 0.99	1.00, 1.50	1.60, 7.10
PFOA	1.39	0.22	0.22, 10.00	0.22, 0.91	0.92, 1.50	1.60, 10.00

NB: PFOS denotes perfluorooctane sulfonate; PFOA denotes perfluorooctanoic acid.

**Table 3:** Results of the analysis treating PFOS and PFOA concentrations as dimensional and categorical measures.

			Intercept		Interaction with age	
			β (SE) *p* value		β (SE) *p* value	
			crude	adjusted	crude	adjusted
Dimensional	PFOS					
	Overall		− 0.27 (0.14) 0.05	− 0.35 (0.14) 0.01	0.0038 (0.002) 0.08	0.0038 (0.002) 0.09
	Boys		0.001 (0.18) > 0.99	− 0.095 (0.19) 0.62	0.0011 (0.003) 0.71	0.0011 (0.003) 0.72
	Girls		− 0.67 (0.2) 0.001	− 0.69 (0.2) 0.001	0.0081 (0.003) 0.015	0.0082 (0.003) 0.014
	PFOA					
	Overall		− 0.22 (0.12) 0.07	− 0.26 (0.13) 0.05	0.0051 (0.002) 0.01	0.0051 (0.002) 0.01
	Boys		0.15 (0.18) 0.4	0.16 (0.19) 0.41	− 0.001 (0.003) 0.72	− 0.001 (0.003) 0.74
	Girls		− 0.59 (0.17) < 0.001	− 0.62 (0.18) < 0.001	0.011 (0.003) < 0.001	0.011 (0.003) < 0.001
Categorical	PFOS					
	Overall	Low	0.00 (Reference)	0.00 (Reference)	0.00 (Reference)	0.00 (Reference)
		Middle	0.042 (0.071) 0.55	0.022 (0.07) 0.75	− 0.0001 (0.0012) 0.91	− 0.0001 (0.0012) 0.92
		High	− 0.16 (0.075) 0.03	− 0.175 (0.076) 0.02	0.0029 (0.0012) 0.02	0.0029 (0.0012) 0.02
	Boys	Low	0.00 (Reference)	0.00 (Reference)	0.00 (Reference)	0.00 (Reference)
		Middle	0.14 (0.10) 0.16	0.11 (0.1) 0.28	− 0.0022 (0.0017) 0.2	− 0.0022 (0.0017) 0.2
		High	− 0.021 (0.11) 0.84	− 0.03 (0.11) 0.75	0.0012 (0.0017) 0.49	0.0012 (0.0017) 0.49
	Girls	Low	0.00 (Reference)	0.00 (Reference)	0.00 (Reference)	0.00 (Reference)
		Middle	− 0.066 (0.096) 0.49	− 0.083 (0.094) 0.38	0.0019 (0.0016) 0.24	0.002 (0.0016) 0.23
		High	− 0.33 (0.10) 0.002	− 0.34 (0.11) 0.002	0.005 (0.0018) 0.005	0.005 (0.0018) 0.004
	PFOA					
	Overall	Low	0.00 (Reference)	0.00 (Reference)	0.00 (Reference)	0.00 (Reference)
		Middle	− 0.091 (0.072) 0.21	− 0.069 (0.072) 0.34	0.0044 (0.0012) < 0.001	0.0044 (0.0012) < 0.001
		High	− 0.16 (0.074) 0.03	− 0.18 (0.078) 0.02	0.0038 (0.0012) 0.002	0.0039 (0.0012) 0.002
	Boys	Low	0.00 (Reference)	0.00 (Reference)	0.00 (Reference)	0.00 (Reference)
		Middle	0.0049 (0.10) 0.96	0.026 (0.1) 0.8	0.0029 (0.0017) 0.08	0.0029 (0.0017) 0.08
		High	0.044 (0.10) 0.67	0.03 (0.11) 0.79	0.0003 (0.0017) 0.88	0.0003 (0.0017) 0.86
	Girls	Low	0.00 (reference)	0.00 (Reference)	0.00 (Reference)	0.00 (Reference)
		Middle	− 0.19 (0.098) 0.06	− 0.17 (0.1) 0.1	0.0059 (0.0016) < 0.001	0.0059 (0.0016) < 0.001
		High	− 0.41 (0.1) < 0.001	− 0.41 (0.11) < 0.001	0.0078 (0.0017) < 0.001	0.0079 (0.0017) < 0.001

Controlling covariates: maternal age at delivery, maternal BMI before pregnancy, maternal education, household income, maternal smoking during pregnancy, parity, gestational age, and duration of breast feeding. NB: SE denotes standard error, PFOS denotes perfluorooctane sulfonate, PFOA denotes perfluorooctanoic acid.

**Figure 2:**
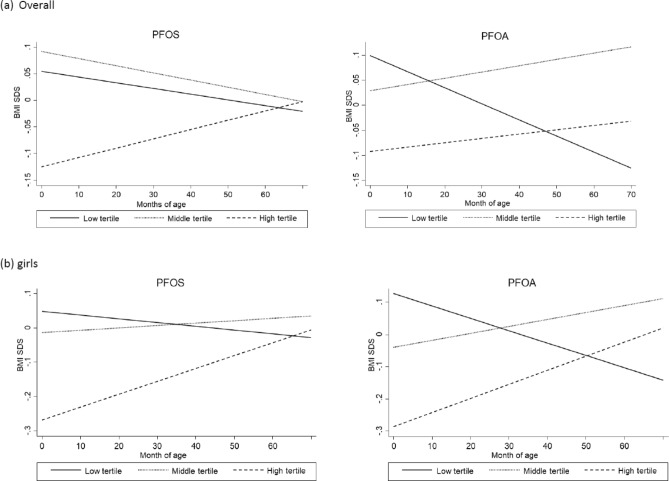
BMI standard deviation scores (SDS) by months of age and tertiles of concentrations of perfluorooctane sulfonate (PFOS) and perfluorooctanoic acid (PFOA) estimated from growth curve modeling analyses adjusted for covariates.

To identify the high risk group by PFAS exposure levels, we repeated the analysis above with categorical measures (low, medium, and high) of PFOS and PFOA concentrations, with the low tertile group set as a reference. Only the high PFOS concentration was associated with lower BMI SDS at birth significantly (Table 3, Fig. 2a). There was a significant positive interaction between the high PFOS concentration and child’s age on BMI SDS, again indicating the greater BMI SDS as they grow (positive slope). Consequently, BMI SDS for the high PFOS concentration was lower than that of the low PFOS concentration from birth to infancy, but gradually increased over time as the child grew and exceeded it at approximately 63 months of age. A similar pattern was found for PFOA. Only the high, but not the middle, PFOA concentration was associated with significantly lower BMI SDS at birth. There was a significant positive interaction between the middle and the high PFOA concentration and child’s age. Here again predected BMI SDS increase in these groups (middle and high) relative to low group as they grow. Furthermore, the predicted BMI SDS was lower in children with the high PFOA concentration than children with the lower concentration in infancy but it increased over time and exceeded it at about 47 months of age among children exposed to high PFOA. In the analysis by sex, only among girls, higher PFOS and PFOA concentrations were associated with lower BMI SDS at birth and had significant positive interactions with child's age on BMI SDS ( i.e., the rate of the increase on BMI SDS is positive as they grow) in both unadjusted and adjusted analyses (Fig. 2b). At about 65 months for PFOS and 50 months for PFOA, BMI SDS of girls with high level of exposure exceeded than that of girls with low level of exposure.

## Discussion

### Principal findings

We prospectively examined the trajectory of BMI SDS in 597 children over 66 months (5½ years) through an examination of the PFAS exposure in UCB serum. There were three main findings. First, prenatal exposure to PFAS was associated with lower BMI SDS during infancy. Second, it was associated with an increase in BMI SDS in later childhood. Although the effect sizes of the interactions between PFAS concentration and child’s age (i.e. the rate of growth in BMI SDS over time) are relatively small in early ages, the actual impact on BMI SDS at 66 months of age is not negligible. For example, an effect size of 0.001 for the interaction (slope) means that 10-time increase in PFAS concentration (due to the log-10 transformation) resulting in 0.66 SD increase in BMI at 66 months of age, which is substantial. Third, girls may be more vulnerable than boys to prenatal exposures to PFAS. These findings are consistent with previous research and support the hypothesis that PFAS may have the initial BMI-lowering effect in early infancy, but the effect could reverse during early childhood and become possible obesity risks.

### Interpretation

Our findings of PFOA are consistent with other studies that reported an association between higher prenatal PFAS exposure and an elevated risk of obesity at 8 years and 20 years of age^21,24,36^. However, our study ameliorated the limitation presented by Chen et al.^28^ that did not include gestational age in covariates and further provided evidence that prenatal exposure to PFOS was associated with lower offspring’s BMI SDS in infancy and then upward trajectory of BMI SDS development as the offspring grow. However, unlike the results by Chen and colleagues, in our study, PFOA was associated with greater BMI SDS later in childhood, especially among girls. Low birth weight itself would lead to obesity in adulthood^37^.

It is important to consider adiposity rebound (AR) when interpreting our findings. AR is generally explained by the BMI increasing rapidly after birth, then starts to decrease at around 1 year, reaching its lowest concentration at 5 to 7 years of age^38^. Our results suggest that higher UCB serum PFAS concentrations may be associated with an earlier onset of AR, which is a well-known predictor of an elevated risk of obesity and metabolic syndrome in adulthood^39–42^. Since reducing child obesity is an ultimate form of prevention for future diseases and reduction in the related social costs, one possible effective prevention can be to increase our surveillance of PFAS in the environment especially during pregnancy and offering early interventions targeted toward those children at a higher risk of obesity by virtue of prenatal PFAS exposure^43^. It is also noteworthy among 599 samples we investigated, in 597 (99.5%) samples we found PFAS residues in UCB, indicating the prenatal exposures to the substances are pervasive. While our findings are preliminary and still require replication, they are alarming and deserve further investigation.

PFAS have an effect on a child’s body weight through different potential mechanisms. First, PFAS bind to PPAR, which are ligand-activated transcription factors and involved in lipid metabolism. The PPAR family was reported to regulate adipogenesis and weight gain, and is affected by chemical compounds such as tributyltin^44^. Both PFOS and PFOA significantly activate PPARα^14^, which regulates lipid metabolism in the liver. Second, PFAS may act as an endocrine disrupter which have effects similar to estrogen^45,46^. Prenatal exposure to environmental estrogens may affect the expression of genes related to obesity via modifying the programming of various estrogen target tissues during early development^47^. In line with this theory, two prior studies reported that the association between prenatal exposure to PFAS and the risk of obesity was stronger among girls than boys, which is consistent with our finidings^24,36^. Third, it is possible that prenatal exposure to PFAS affects body weight via a change in thyroid function^10^, although there are a limited number of studies, and the results are inconclusive. As hypothyroidism is known to decrease prenatal weight gain^48^, it can be an underlying mechanism for decreased BMI during infancy. However, this does not explain the increase in BMI SDS during later childhood. Last, maternal serum concentrations of PFAS in pregnancy were reported to be inversely associated with UCB glucocorticoid, and may lead to lower birthweight^11^. However, we found no notable associations between these two variables in our samples.

In our study, the participants’ year of birth (from 2007 to 2012) was associated with decreased concentrations of PFAS (β =  − 0.28, 95% CI − 0.35, − 0.21 for PFOS, β =  − 0.26, 95% CI − 0.33, − 0.20 for PFOA). This is consistent with previous studies, which reported that concentrations of PFOS and PFOA in human serum peaked c. 2000 and subsequently continued to decrease^49^. This would be attributable to the global phase-out of production of these compounds in the early 2000s. However, the results remained unchanged after adding the year of birth in the model. As stated above, almost all of our participants (99.5%) were exposed to PFAS despite that they were born later than 2000. This strongly urges policy makers and researchers to keep monitoring the PFAS levels in the environment.

### Strengths of the study

The current study had several strengths. First, the study is a longitudinal study of our established cohort, with prospective observation and repeated measures from birth to 66 months of age with low attrition rates over time. With such a study design, the valid evaluation of longitudinal changes of BMI instead of reporting one snapshot of BMI in childhood is possible. Having multiple assessment points further allows us to use more advanced and sophisticated statistical methods that help uncover the complexity of the outcome among children at greater risk for obesity in childhood – the group that did not show initial problems with excessive weight gain during infancy. Moreover, using umbilical cord serum, not maternal serum, samples enables direct assessment of prenatal PFAS exposure among offspring.

### Limitations of the study

The study also has several limitations. First, we have only assessed the cohort for their BMI up to 66 months (5½ years) of age. Optimally, we would have longitudinal observation beyond 5½ years of age because the mean age of AR is around 6 years of age. Second, measuring equipment for weight and height was not unified across each measurement. Also, measurement errors could have been minimized if the measuring equipment had been unified. Third, the children’s exposure to PFAS might not have been limited to exposure in the prenatal period. Postnatal exposures to PFAS, such as through breast milk, drinking water, diet, and dust inhalation, could also be possible. Last, although BMI in childhood can be affected by dietary habits^50^, no variable reflecting dietary habits was incorporated in our analyses.

## Conclusions

Our findings demonstrate that the effect of prenatal exposure to PFAS is a possible risk for obesity through an increase in BMI SDS, especially for girls. As PFAS are widely detected in lifestyle choices and the environment, it is important to understand the health effects of PFAS as well as their pathways and mechanisms to suboptimal health outcomes during childhood.

## Supplementary Information

Supplementary Information.

## References

[1] Rahman MF, Peldszus S, Anderson WB (2014). Behaviour and fate of perfluoroalkyl and polyfluoroalkyl substances (PFASs) in drinking water treatment: A review. Water Res..

[2] Mariussen E (2012). Neurotoxic effects of perfluoroalkylated compounds: Mechanisms of action and environmental relevance. Arch. Toxicol..

[3] Fromme H, Tittlemier SA, Volkel W, Wilhelm M, Twardella D (2009). Perfluorinated compounds–exposure assessment for the general population in Western countries. Int. J. Hyg. Environ. Health.

[4] Lemal DM (2004). Perspective on fluorocarbon chemistry. J. Org. Chem..

[5] Buck RC (2011). Perfluoroalkyl and polyfluoroalkyl substances in the environment: Terminology, classification, and origins. Integr. Environ. Assess. Manag..

[6] Butenhoff JL, Olsen GW, Pfahles-Hutchens A (2006). The applicability of biomonitoring data for perfluorooctanesulfonate to the environmental public health continuum. Environ. Health Perspect..

[7] Olsen GW (2007). Half-life of serum elimination of perfluorooctanesulfonate, perfluorohexanesulfonate, and perfluorooctanoate in retired fluorochemical production workers. Environ. Health Perspect..

[8] D'Eon JC, Simpson AJ, Kumar R, Baer AJ, Mabury SA (2010). Determining the molecular interactions of perfluorinated carboxylic acids with human sera and isolated human serum albumin using nuclear magnetic resonance spectroscopy. Environ. Toxicol Chem..

[9] Inoue K (2004). Perfluorooctane sulfonate (PFOS) and related perfluorinated compounds in human maternal and cord blood samples: Assessment of PFOS exposure in a susceptible population during pregnancy. Environ. Health Perspect..

[10] Ballesteros V (2017). Exposure to perfluoroalkyl substances and thyroid function in pregnant women and children: A systematic review of epidemiologic studies. Environ. Int..

[11] Goudarzi H (2017). The association of prenatal exposure to perfluorinated chemicals with glucocorticoid and androgenic hormones in cord blood samples: The Hokkaido study. Environ. Health Perspect..

[12] Hines EP (2009). Phenotypic dichotomy following developmental exposure to perfluorooctanoic acid (PFOA) in female CD-1 mice: Low doses induce elevated serum leptin and insulin, and overweight in mid-life. Mol. Cell. Endocrinol..

[13] Shi Z (2009). The effect of perfluorododecanonic acid on endocrine status, sex hormones and expression of steroidogenic genes in pubertal female rats. Reprod Toxicol.

[14] Takacs ML, Abbott BD (2007). Activation of mouse and human peroxisome proliferator-activated receptors (alpha, beta/delta, gamma) by perfluorooctanoic acid and perfluorooctane sulfonate. Toxicol. Sci..

[15] Lau C (2006). Effects of perfluorooctanoic acid exposure during pregnancy in the mouse. Toxicol. Sci..

[16] Wolf CJ (2007). Developmental toxicity of perfluorooctanoic acid in the CD-1 mouse after cross-foster and restricted gestational exposures. Toxicol. Sci..

[17] Apelberg BJ (2007). Cord serum concentrations of perfluorooctane sulfonate (PFOS) and perfluorooctanoate (PFOA) in relation to weight and size at birth. Environ. Health Perspect..

[18] Chen MH (2012). Perfluorinated compounds in umbilical cord blood and adverse birth outcomes. PLoS ONE.

[19] Fei C, McLaughlin JK, Tarone RE, Olsen J (2007). Perfluorinated chemicals and fetal growth: A study within the Danish National Birth Cohort. Environ Health Perspect.

[20] Maisonet M (2012). Maternal concentrations of polyfluoroalkyl compounds during pregnancy and fetal and postnatal growth in British girls. Environ. Health Perspect..

[21] Braun JM (2016). Prenatal perfluoroalkyl substance exposure and child adiposity at 8 years of age: The HOME study. Obesity (Silver Spring).

[22] Gyllenhammar I (2018). Perfluoroalkyl acid levels in first-time mothers in relation to offspring weight gain and growth. Environ Int.

[23] Høyer BB (2015). Anthropometry in 5- to 9-year-old Greenlandic and Ukrainian children in relation to prenatal exposure to perfluorinated alkyl substances. Environ Health Perspect..

[24] Mora AM (2017). Prenatal Exposure to Perfluoroalkyl Substances and Adiposity in Early and Mid-Childhood. Environ Health Perspect.

[25] Andersen CS (2013). Prenatal exposures to perfluorinated chemicals and anthropometry at 7 years of age. Am. J. Epidemiol..

[26] Barry V, Darrow LA, Klein M, Winquist A, Steenland K (2014). Early life perfluorooctanoic acid (PFOA) exposure and overweight and obesity risk in adulthood in a community with elevated exposure. Environ. Res..

[27] Liu, P., Yang, F., Wang, Y. & Yuan, Z. Perfluorooctanoic Acid (PFOA) Exposure in Early Life Increases Risk of Childhood Adiposity: A Meta-Analysis of Prospective Cohort Studies. *Int. J. Environ. Res. Public Health* **15**. 10.3390/ijerph15102070 (2018). 10.3390/ijerph15102070 PMC6209901 30241417

[28] Chen MH (2017). The impact of prenatal perfluoroalkyl substances exposure on neonatal and child growth. Sci. Total Environ..

[29] Takagai S (2016). Cohort profile: Hamamatsu birth cohort for mothers and children (HBC Study). Int J Epidemiol.

[30] Nishimura T, Takei N, Tsuchiya KJ, Asano R, Mori N (2016). Identification of neurodevelopmental trajectories in infancy and of risk factors affecting deviant development: A longitudinal birth cohort study. Int J. Epidemiol..

[31] Tsuchiya KJ (2010). Searching for very early precursors of autism spectrum disorders: The Hamamatsu Birth Cohort for Mothers and Children (HBC). J. Dev. Orig. Health Dis..

[32] Kato N, Takimoto H, Sudo N (2011). The cubic functions for spline smoothed L, S and M values for BMI reference data of Japanese children. Clin Pediatr Endocrinol.

[33] Arbuckle JL, Schumacker G (1996). Full information estimation in the presence of incomplete data. Advanced structural equation modeling.

[34] Enders CK, Bandalos DL (2001). The Relative Performance of Full Information Maximum Likelihood Estimation for Missing Data in Structural Equation Models. Struct. Equ. Model..

[35] McArdle JL, Hamagami JL, Marcoulides G, Schumacker R (1996). Full information estimation in the presence of incomplete data. Advanced structural equation modeling techniques.

[36] Halldorsson TI (2012). Prenatal exposure to perfluorooctanoate and risk of overweight at 20 years of age: A prospective cohort study. Environ Health Perspect..

[37] Barker D (2004). The developmental origins of chronic adult disease. Acta Paediatr..

[38] Rolland-Cachera MF (1984). Adiposity rebound in children: A simple indicator for predicting obesity. Am. J. Clin. Nutr..

[39] Whitaker RC, Pepe MS, Wright JA, Seidel KD, Dietz WH (1998). Early adiposity rebound and the risk of adult obesity. Pediatrics.

[40] Williams SM, Goulding A (2009). Patterns of growth associated with the timing of adiposity rebound. Obesity (Silver Spring).

[41] Eriksson JG, Forsen T, Tuomilehto J, Osmond C, Barker DJ (2003). Early adiposity rebound in childhood and risk of Type 2 diabetes in adult life. Diabetologia.

[42] Koyama S (2014). Adiposity rebound and the development of metabolic syndrome. Pediatrics.

[43] Pigeot I, de Henauw S, Baranowski T (2015). The IDEFICS (Identification and prevention of Dietary- and lifestyle-induced health effects in children and infants) trial outcomes and process evaluations. Obes. Rev..

[44] Grun F (2006). Endocrine-disrupting organotin compounds are potent inducers of adipogenesis in vertebrates. Mol. Endocrinol..

[45] Zhao Y (2012). Perfluorooctanoic acid effects on ovaries mediate its inhibition of peripubertal mammary gland development in Balb/c and C57Bl/6 mice. Reprod Toxicol.

[46] Liu C, Du Y, Zhou B (2007). Evaluation of estrogenic activities and mechanism of action of perfluorinated chemicals determined by vitellogenin induction in primary cultured tilapia hepatocytes. Aquat. Toxicol. (Amsterdam, Netherlands).

[47] Newbold RR, Padilla-Banks E, Jefferson WN, Heindel JJ (2008). Effects of endocrine disruptors on obesity. Int. J. Androl..

[48] Karakosta P (2012). Thyroid dysfunction and autoantibodies in early pregnancy are associated with increased risk of gestational diabetes and adverse birth outcomes. J. Clin. Endocrinol. Metab..

[49] D'Eon JC, Mabury SA (2011). Is indirect exposure a significant contributor to the burden of perfluorinated acids observed in humans?. Environ Sci Technol.

[50] Emmett PM, Jones LR (2015). Diet, growth, and obesity development throughout childhood in the Avon Longitudinal Study of Parents and Children. Nutr. Rev..

