# Supplementary material for: Umbilical cord serum concentrations of perfluorooctane sulfonate, perfluorooctanoic acid, and the body mass index changes from birth to 5 1/2 years of age
Source: Sci Rep. 2021 Oct 5;11:19789. doi: 10.1038/s41598-021-99174-3 (PMC8492859; doi:10.1038/s41598-021-99174-3)
Supplement: Supplementary file 1 — Supplementary Information. [file 41598_2021_99174_MOESM1_ESM.docx]

**Supplementary information**

**The details of assay method**

・Pretreatment method

2ng of internal standard was added to 0.5mL of serum sample, then 1mL of methanol was added in order to remove protein. Following that, 10mL of water and 0.1mL of formic acid were added. The supernatant of this sample solution was load to solid phase (Oasis WAX). Then the target substances were eluted by 5mL of methanol containing 0.1% ammonia. The eluent was concentrated to less than 0.5 mL using gentle stream of nitrogen gas. Measurement solution was obtained after adjusting to 0.5mL of sample volume.

・Measurement

PFOS, PFOA were measured with a liquid chromatograph tandem mass spectrometer (LC/MS/MS). The measurement condition was shown in Table 1.

**Supplementary Table 1.** LC/MS/MS measurement condition for perfluoro compounds (PFCs)

| Equipment | LC：LC-20A Prominence （Shimadzu Corporation） | | | |
| --- | --- | --- | --- | --- |
|  | MS：API 4000 (AB SCIEX) | | | |
| LC conditions |  | | | |
| Column | Inertsil ODS-SP (2.1 mm(i.d.)×150 mm、3μm) (GL Sciences) | | | |
| Mobile phase A | 10mM ammonium acetate | | | |
| Mobile phase B | acetonitrile | | | |
| Gradient | 0-2 min | A：65% | | B：35% |
|  | 2-8 min | A：65→50% | | B：35→50% |
|  | 8-10.5 min | A：50% | | B：50% |
|  | 10.5-15.5 min | A：50→20% | | B：50→80% |
|  | 15.5-20.5 min | A：20% | | B：80% |
|  | 20.5-30 min | A：65% | | B：35% |
| Mobile phase flow rate | 0.2 mL/min | | | |
| Column temperature | 40°C | | | |
| Sample injection volume | 10μL | | | |
| MS/MS condition |  | | | |
| Ionization method | ESI Negative (MRM：Multiple Reaction Monitoring) | | | |
| Monitor ion（*m/z*） |  | | | |
| Measured substance | For quantification | | For confirmation | |
| PFOS | 498.9→80.0 | | 498.9→99.0 | |
| PFOA | 412.9→369.0 | | 412.9→169.0 | |
| Internal standard substance |  | |  | |
| *^13^C_4_*-PFOS | 502.9→80.0 | | 502.9→99.0 | |
| *^13^C_4_*-PFOA | 416.9→372.0 | | 416.9→169.0 | |

・Validation of this method

1) Calibration curve

The calibration curve ranged from 0.01 to 30 ng/mL with more than 0.999 coefficient of determination (r2) value.

2)Calculation of Instrumental Detection Limit (IDL)

The standard solution with the lowest concentration used for the calibration curve was repeatedly measured 7 times. The measured values were calculated according to the “Guidelines for conducting a survey on the environment of chemical substances (2015)”, which applied the definition of Currie (1997).

3)Calculation of Method Detection Limit (MDL) and Method Quantification Limit (MQL)

A test solution in which the internal standard and the native standard of about 5 times of IDL were added to a blank sample was pretreated in the same method as the actual sample.

This operation was repeated 7 times, and MDL and MQL were calculated according to the definition of Currie (1997) as in IDL. MDL were 3pg / mL for both PFOS and PFOA.

4)Recovery

A test solution in which the internal standard and 20 pg native standard were added to a blank sample was subjected to the same pretreatment as the actual sample. This procedure was repeated 7 times, and the range of recovery ratio for PFOS was 68.4 to 113.2%, and for PFOA was 67.8 to 109.2%.

・Round robin test

Participated in G-EQUAS 59, an international round robin test and the result was within the tolerance for the analysis of PFCs in serum.

| Variables | Included n=597 Median (IQR) | Excluded n=661 Median (IQR) | P-value |
| --- | --- | --- | --- |
| Maternal age at birth (years) | 31.8 (28.1, 35.3) | 31.4 (27.9, 35.0) | 0.21 |
| Maternal pre-pregnancy BMI (kg/m2) | 20.3 (18.8, 22.5) | 20.1 (18.8, 21.8) | 0.12 |
| Household income (10000yen/year) | 550 (420, 700) | 542 (420, 704) | 0.87 |
| Maternal education (years) | 14 (12, 16) | 14 (12, 15) | 0.28 |
| Gestational age (weeks) | 39.11 (38.1, 40.0) | 39.1 (38.0, 40.0) | 0.3 |
| Birthweight (g) | 2982 (2699, 3246) | 2932 (2654, 3212) | 0.05 |
| Duration of breastfeeding (months) | 10 (4, 14 ) | 10 (6, 14) | 0.54 |

**Supplementary Table 2.** Comparison of demographic characteristics between those included and those excluded in Mann-Whitney U-test.

| Variables | Included n=597 | Excluded n=661 | P-value |
| --- | --- | --- | --- |
| Boys | 310 (51.9%) | 338 (51.1%) | 0.78 |
| Primiparous | 274 (45.9%) | 352 (53.3%) | 0.009 |
| Smoking during pregnancy | 41 (6.9%) | 63 (9.5%) | 0.09 |

**Supplementary Table 3.** Comparison of demographic characteristics between those included and those excluded in χ^2^ test.

The observed marginally significant difference in birth weight between those included and excluded in this study may due to the relatively high rate of failure in cord blood sampling in low birth weight infants. The reason for significantly low rate of primiparous children among those included is unknown.

・References

L.A. Currie (1997), Detection: International update, and some emerging di-lemmas

involving calibration, the blank, and multiple detection decisions. Chemometrics and

Intelligent Laboratory Systems, 37: 151-181 (http://www.chemometry.com/Index/Links

and downloads/Papers/Currie, chemolab, 37 (1997) 151.pdf)
